# Artificial intelligence-supported therapeutic interventions for autism spectrum disorder: a systematic review

**DOI:** 10.3389/fpsyt.2026.1833853

**Published:** 2026-08-28

**Authors:** Julia Kuca, Magdalena Stencel, Błażej Pilarski, Szymon Florek, Robert Pudlo

**Affiliations:** 1 Student’s Research Group, Department of Psychiatry, Faculty of Medical Sciences in Zabrze, Medical University of Silesia in Katowice, Tarnowskie Góry, Poland; 2 Department of Psychoprophylaxis, Faculty of Medical Sciences in Zabrze, Doctoral School, Medical University of Silesia in Katowice, Tarnowskie Góry, Poland; 3 Department of Psychoprophylaxis, Faculty of Medical Sciences in Zabrze, Medical University of Silesia in Katowice, Tarnowskie Góry, Poland

**Keywords:** artificial intelligence, autism spectrum disorder, intervention, machine learning, social robotics, systematic review, virtual reality, wearables

## Abstract

**Background:**

The increased prevalence of ASD has generated a pressing demand for flexible therapeutic and educational tools. AI has been suggested as a potential bridge to this gap, but the translation from a model to a clinical application necessitates rigorous assessment. The purpose of the present review is to compile and examine the existing literature to demonstrate AI interventions that have advanced from a proposed model to being used with human participants.

**Methods:**

We systematically searched five databases (Embase, PubMed, ScienceDirect, IEEE Xplore, Web of Science; Jan 2016–Dec 2025) for AI-based ASD interventions. Two reviewers independently assessed eligibility. Inclusion criteria were as followed: (1) participants with confirmed ASD diagnoses; (2) an intervention sample size of N ≥ 6; (3) AI as a central therapeutic, educational, or rehabilitative component; and (4) multi-session protocols with specified timeframes. Study types ranged from system development and feasibility trials to RCTs.

**Results:**

14 studies met inclusion criteria. AI (e.g. robotics, VR, and wearables) functioned as a social mediator, improving social-emotional outcomes (e.g., ADOS, SRS scores) by reducing cognitive load. Significant mechanisms included real-time task adaptation and precise behavioral monitoring via e.g. eye-tracking. However, significant heterogeneity was observed in intervention dosage (median 4–12 hours). Most studies were limited by small, male-dominated samples (N < 20) and a total absence of adult participants.

**Conclusion:**

Based on the findings, AI seems highly promising for patient-specific ASD therapy via proactive, data-driven scaffolding. In order to move toward implementation of AI in ASD care, more RCTs and crucial augmentation of representation gaps concerning adult and female ASD phenotype studies are required.

## 1 Introduction

Autism Spectrum Disorder (ASD) is described in literature as a neurodevelopmental disorder characterised by persistent difficulties in communication and social interaction, as well as the occurrence of restricted and repetitive patterns of behavior, interests or activities (1, 2). The diagnosis of ASD is based on the diagnostic criteria contained in the DSM-5-TR (2013/2022) and ICD-11 (2019) classifications, in which the disorder is conceptualized as a spectrum with a continuous nature of symptom severity. There are three levels of support: from Level 1, which includes individuals requiring relatively little assistance, to Level 3, associated with the need for intensive support and significant adaptive difficulties (2, 3). Due to limitations in cognitive capabilities, linguistic and sensory issues, many autistic individuals are unable to function independently in daily life or in the labor market (especially when their needs are not met). Moreover, healthcare and therapeutic service costs are greater for persons with ASD than for the general population. ASD affects approximately 1-2% of the world’s population, with a higher prevalence in children according to World Health Organization 2023 (2, 4, 5).

Various therapeutic methods are employed in working with individuals on the autism spectrum, such as Applied Behavior Analysis (ABA), the Early Start Denver Model (ESDM), Picture Exchange Communication Systems (PECS), and Discrete Trial Training (DTT) (6). While the effectiveness of ABA therapy has been confirmed in numerous studies, an increasing number of neurodiversity researchers express concerns regarding its application (7). Naturalistic developmental behavioral interventions, such as Pivotal Response Treatment (PRT)—which is a variation of the ABA approach—are also utilized (8). Despite their efficacy, the diversity of these methods remains inaccessible to many due to the multidisciplinary nature of services and high costs; furthermore, some researchers question their effectiveness due to the inherent heterogeneity of ASD (6). Speech-language interventions and developmental communication support programs, including naturalistic approaches and personalized trainings designed to help caregiver-child interactions, are also critical aspects of therapeutic interventions and have been associated with enhanced linguistic and communicative competence in many subjects (9). In education, numerous strategies are implemented to support cognitive and social development, aiming for the full participation of students with ASD within the school environment (10).

Artificial Intelligence (AI) is a field of computer science that integrates diverse disciplines to create systems capable of performing tasks typically associated with human intelligence, such as learning from experience, reasoning, and decision-making (11, 12). A key approach within AI is machine learning, which involves designing algorithms that, rather than relying on rigidly defined rules, learn patterns directly from data to make predictions or classify new cases (13). A specialized role is played by deep learning—an advanced form of machine learning utilizing multi-layered neural networks. This enables the effective analysis of large and complex datasets, such as medical images, signals, or text, finding broad applications in areas like diagnostics and signal processing (14). In psychiatry, AI systems play an increasing role in monitoring disease progression, assisting in diagnosis, and providing therapeutic support tools (e.g., speech and behavior analysis, or data from mobile devices), which opens possibilities for more objective and continuous tracking of a patient’s mental state (15). In the context of neurodevelopmental disorders, including ASD, research indicates that machine learning and deep learning models can support early detection, automated behavior analysis from video recordings, processing of linguistic features, and the analysis of biomedical signals, although practical application requires data standardization and further clinical validation (16, 17).

In recent years, a dynamic increase in the number of publications concerning AI applications in ASD has been observed, leading to fragmentation and difficulties in synthesizing the available evidence. Previous reviews have examined selected categories of technological interventions for autism, including socially assistive robots (18), virtual reality (VR) (19), mobile and ICT-assisted interventions (20, 21), and assistive technologies for various neurodevelopmental disorders (22), rather than focusing solely on autism. However, these reviews have typically focused on specific technological modalities in isolation or have addressed broader technologically assisted interventions without a specific emphasis on AI-based approaches. Existing literature reviews are predominantly narrative, focusing primarily on diagnostics, or are narrow and outdated in scope. Consequently, they fail to provide a systematic assessment of AI-based interventions. Therefore, a clear research gap exists, justifying the need for an up-to-date, comprehensive systematic review focused exclusively on AI-driven interventions in ASD. The literature is characterized by significant methodological heterogeneity, a limited number of randomized controlled trials (RCTs), and discrepancies regarding populations, interventions, and outcome measures, all of which hinder the comparability of results. Additionally, significant challenges are posed by ethical issues (data privacy protection, informed consent, algorithmic biases) and the limited feasibility of implementing research findings into clinical practice. The objective of this systematic review is to evaluate the current state of knowledge regarding the application of artificial intelligence-based interventions in individuals with autism spectrum disorders.

## 2 Methods

### 2.1 Search strategy

A systematic search of five electronic databases (ScienceDirect, PubMed, Embase, Web of Science, and IEEE Xplore) was performed independently by two authors. The search, covering all records up from year 2016 to December 10, 2025, utilized both MeSH terms and keywords related to children, adolescents and adults with ASD to capture all relevant studies (“autism” or “autism spectrum disorder”) AND (“therapy” or “intervention” or “training”) AND (“Artificial Intelligence” or “machine learning” or “virtual reality” or “VR” or “robot” or “augmented reality “ or “app”). Included papers range from 2016 to 2025. We screened studies only published in English.

### 2.2 Design and eligibility criteria

The review was conducted following the Preferred Reporting Items for Systematic Reviews and Meta-analyses (PRISMA) (23). PICOS (Population, Intervention, Comparator, Outcomes, Study Design) criteria were used to select studies, as outlined in **Table 1** (24).

**Table 1:** PICOS criteria for inclusion of studies.

Parameter	Criteria
Population	Children, adolescents and adults with formal ASD diagnosis
Intervention	Any therapeutic, educational, or rehabilitative intervention with Artificial Intelligence (AI) as a core component
Comparator	No limitations on control measures
Outcomes	Divided into 3 domains: social, cognitive, emotion
Study design	RCTs, quasi-experimental, single-group pre-post

Inclusion criteria: (P) children, adolescents and adults with formal ASD diagnosis (including DSM-IV/V, ADOS-2) from a minimum of 6 people in the intervention group, (I) involving any therapeutic, educational, or rehabilitative intervention with Artificial Intelligence (AI) as a core component e.g.: Socially Assistive Robots, AI-driven virtual reality agents, intelligent tutoring systems (ITS), chatbots and wearables. The modalities of AI applied in the reviewed interventions go beyond neural network techniques. The reviewed research involved the use of assistive technologies based on the Narrow AI framework. In particular, ML classifiers were used in the Google Glass and CognitiveBotics program; Large Language Models (LLM) were utilized via ChatGPT; and Convolutional Neural Networks (CNN) were deployed within the web-based system ‘EmoLand’ and the application-based system ‘FECTS.’ Furthermore, a machine learning-based sensory management recommendation system (SMRS) integrated Gradient Boosting Decision Trees (GBDT) and Random Forests (RF) within its application framework. Within serious game interventions, ML applications utilized the proximal algorithm for dual dictionaries learning (PADDLE). Finally, the Virtual Reality-based OpenSimulator platform and humanoid NAO robots reviewed in the literature were driven by Rule-Based AI systems.

Studies were included if there was more than one intervention session with reported duration, and they were performed on different days; (C) studies such as treatment as usual, waiting list control, active control (non-AI) and no control; (O) Utilizing standardized assessment instruments to track behavioral changes and providing clear change-score data to support evidence-based reviews; (S) RCTs, NRCTs or quasi-experimental and single-group pre-post studies were included.

Exclusion criteria: Participants with “autistic traits” or self-diagnosed individuals without clinical confirmation. Studies that focus solely on other neurodevelopmental disorders (e.g., ADHD, Down Syndrome) without a separately analyzed ASD subgroup. The following studies were excluded from the review: those involving interventions in which AI is used solely for diagnosis (screening), rather than for therapy or training; studies where the scope was limited solely to program design or system implementation; and studies where the use of AI was restricted solely to data collection and analytical processing, without the need to generate interactive, real-time adaptations during the intervention. We also excluded individual case studies and conference abstracts without the full text.

**Figure 1:**
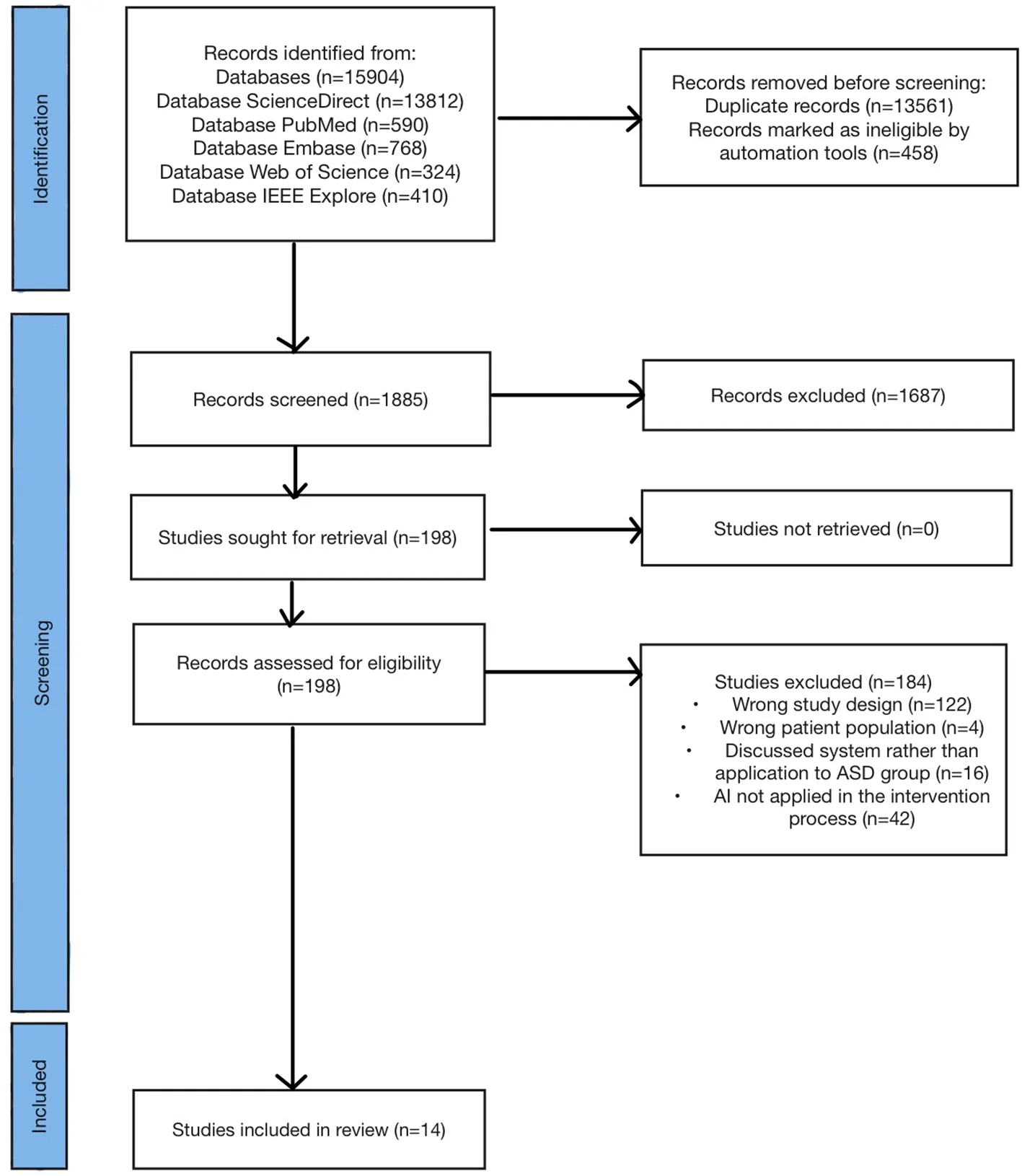
PRISMA flow diagram.

PRISMA study selection flowchart is presented in **Figure 1**.

### 2.3 Literature screening and data extraction

Records were managed in Rayyan, where duplicates were removed before two authors independently screened titles and abstracts. Studies meeting the initial criteria underwent a full-text review to determine final inclusion. We also manually screened the reference lists of included studies to identify additional relevant works. Data extraction was then conducted by three authors using a structured template to capture information such as baseline characteristics and intervention details, e.g. type of technology, AI algorithms, intervention domain, and theoretical framework. Quality assessment was conducted via critical narrative synthesis due to study heterogeneity.

Baseline characteristics of reviewed studies are presented in **Table 2**.

**Table 2:** Baseline characteristics.

Author (year)	Type of study	SG - gender	CG - gender	Mean age/SD - SG	Mean age/SD - CG	CG - ASD/TD	Diagnosis criteria	Level of functioning
Helen M. Genova et al. (2021) (25)	RCT	7M/0F	6M/1F	17.29	17.29	ASD	Formal ASD diagnosis	ASD Level 1/2 support requirement
Horace H.S. Ip et al. (2018) (26)	Empirical intervention study using a waitlist control design	43M/4F	43M/4F	8.92	8.73	ASD	Formal ASD diagnosis	ASD Level 1/2 support requirement
Jewoong Moon et al. (2025) (27)	Exploratory mixed-methods research design	7M/0F	N/A	15.2 (range 13-17)	N/A	N/A	Formal ASD diagnosis	ASD Level 1/2 support requirement
Lingling Deng et al. (2022) (28)	System development and real-life evaluation study	24M/6	24M/6	4.3	4.4	TD	Formal ASD diagnosis	ASD Level 1/2 support requirement
Uğur Aydemir et al. (2025) (29)	Pre-post feasibility study	7M/6F	8M/5F	14.38 (3.06)	13.92 (3.52)	ASD	DSM-V	ASD Level 2/3 support requirement
Voss, C. et al. (2019) (30)	RCT	37M/3F	16M/15F	8.74 (1.79)	8.63 (2.52)	ASD	DSM-IV/DSM-V, SCQ	ASD Level 1/2 support requirement
Harini Atturu et al. (2025) (31)	12-month longitudinal observational study	33M/2F	3M/2F	3.62	3.72	ASD	CARS	ASD Level 1/2 support requirement
Stefano Piana et al. (2021) (32)	Evaluation study	9M/1F	4M/1F	9.6	9.8	ASD	Formal ASD diagnosis	ASD Level 1/2 support requirement
Min Fan et al. (2025) (33)	Research through design and field evaluation	10M/2F	N/A	6.08 +/- 1.24 (range 4 to 7)	N/A	N/A	DSM-V	ASD Level 1/2 support requirement
Guobin Wan et al. (2022) (34)	Preliminary intervention study	10M/2F	N/A	range 3-8	N/A	N/A	DSM-V, CARS	ASD Level 1/2 support requirement
Brian Scassellati et al. (2018) (35)	Single-subject withdrawal design, open-label pilot	7M/5F	NA	9.02 (1.41)	NA	NA	ADI-R, ADOS	ASD Level 1/2 support requirement
Flavia Marino et al. (2020) (36)	RCT	6M/1F	6M/1F	6.08	6.83	ASD	DSM-V, ADOS-2	ASD Level 1/2 support requirement
Eva Yin-han Chung (2019) (37)	Single-group repeated-measures design	14M/0F	N/A	range 9-11	N/A	N/A	DSM-V, SRS	ASD Level 1/2 support requirement
Eva Yin-han Chung et al. (2025) (38)	RCT	54M/6F	20	7.72 (1.42)	N/A	ASD	ADOS, SRS	ASD Level 1/2 support requirement

ADI-R, Autism Diagnostic Interview – Revised; ADOS, Autism Diagnostic Observation Schedule, Second Edition; ASD, Autism Spectrum Disorder; CARS, Childhood Autism Rating Scale; CG, Control Group; DSM, Diagnostic and Statistical Manual of Mental Disorders; F, Female; M, Male; N/A, Not applied; RCT, Randomized Control Trial; SCQ, Social Communication Questionnaire; SG, Study Group; SRS, Social Responsiveness Scale; TD, Typically Developed.

### 2.4 Ethics statement

Ethical review and approval was not required for this study, as it is a systematic review of previously published data. All primary studies included in this review were conducted in accordance with the ethical standards of the respective institutional review boards, as described in the original publications.

## 3 Results

Results were categorized into three domains based on the type of intervention: social, cognitive, and emotional domain. The social domain included interventions like job interview skills, eye contact and joint attention, verbal and non-verbal communication, and gesture imitation. The cognitive domain reported interventions focusing on on-task attention, daily adaptive skills (e.g., motor skills), symbol use, pattern recognition, and educational outcomes. The emotion domain focuses on participants’ ability to recognize, understand, and express their emotions in a safely designed setting. To provide a clearer overall view of the selected studies non-parametric descriptive statistics (medians and interquartile ranges) were calculated for the intervention parameters. These characteristics are detailed in **Table 3**. Details of the interventions and their corresponding results are presented in **Tables 4A–C**.

**Table 3:** Domain characteristics of reviewed studies.

Variable	Total (N=14)	Social domain (N=8)	Cognitive domain (N=4)	Emotion domain (N=6)
Sample size	13 [10-35]	33 [13-44]	22 [10-33]	12 [10-40]
Age of participants (years)	9 [6-10]	9 [6-10]	9 [4-15]	7 [6-9]
Number of sessions	12 [9-16]	14 [12-26]	11 [6-554]	12 [5-13]
Intervention duration (hours)	7 [3-10]	7 [4-11]	9 [5-187]	4 [3-5]

Median [Q1 - Q3].

**Table 4a:** Cognitive domain of reviewed studies.

Study	Type of technology	Role of AI	Intervention - SG	Duration of intervention	Total duration of intervention (hours) per participant	Assessments	Main results	Main outcomes	Acceptability of used system
Jewoong Moon et al. (2025) (27)	VR (OpenSimulator platform)	Diagnostic and progress monitoring system	VR-based scientific problem-solving quests in two modules	6 - 12 sessions	range 6-12	Scavenger Hunting Game, Ball Shooting Game, WCST	Ball-Shooting: predicted Pattern Development (28.5% of variance, p = 0.05). Captured Attention-Switching: longer completion times correlated with cognitive shifting rigidity (r = 0.323, p = 0.051).	The following tasks showed a significant positive relationship with specific representational flexibility skills	High acceptability
Lingling Deng et al. (2022) (28)	Wearable sensors (Apple Watch, Arduino), SMRS app	Assistant Supplement, Behavioral Monitoring System, Adaptive Learning Tool	Session in the classrooms with a teacher and participant with an Apple Watch and GSR sensor. AI recommended physical or environmental adjustments	3 sessions for 30 minutes each (No-SMRS, SMRS session 1 and 2)	1	C-TRF, SUS, Heart rate, GSR, hand movements, and environmental data, AI-driven sensors	C-TRF: significant improvements in the ASD group between the baseline and the intervention phase across all behavioral parameters (p < 0.001).	Reduction in behavior problem scores - improved attention and reduced stress during class	High acceptability
Uğur Aydemir et al. (2025) (29)	LLM (ChatGPT version 4.0)	Assistant Supplement, Behavioral Monitoring System, Adaptive Learning Tool	Parents implemented home physical activity sessions, following AI-recommended programs	One session for 40-minutes, 12 sessions	8	LTEQ	The ChatGPT-delivered intervention group reported an increase in physical activity scores from pre-test (6.69) to post-test (34). Significant group effect (F1, 24 = 108.769; P<.05, ηp2=.819)	Significant improvement in physical activity scores	High acceptability
Harini Atturu et al. (2024) (31)	The CognitiveBotics platform	Assistant Supplement, Behavioral Monitoring System, Adaptive Learning Tool	The four primary learning modalities: Audiovisual Stimulation, Chatbot, AI Interactive Games, Parent Training Videos	One session for 20 minutes, 3 sessions/day for 365 days	365	CARS, VSMS, DST, REEL	The AI-driven adaptive learning platform generated improvements from baseline to the 12-month mark, showing a highly significant increase in mean CARS scores (p < 0.001).	Improvements in cognitive functioning - Developmental Age and Quotient, Receptive and Expressive Language Age	High acceptability

AI, Artificial Intelligence; CARS, Childhood Autism Rating Scale; C-TRF, Caregiver-Teacher Report Form; DST, Developmental Screening Test; GSR, Galvanic Skin Response; LLM, Large Language Model; LTEQ, Leisure Time Exercise Questionnaire; REEL, Receptive Expressive Emergent Language Test; SG, Study Group; SMRS, Sensory Management Recommendation System; SUS, System Usability Scale; VR, Virtual Reality; VSMS, Vineland Social Maturity Scale; WCST, Wisconsin Card Sorting Test.

**Table 4b:** Social domain of reviewed studies.

Study	Type of technology	Role of AI	Intervention - SG	Duration of intervention	Total duration of intervention (hours) per participant	Assessments	Main results	Main outcomes	Acceptability of used system
Helen M. Genova et al. (2021) (25)	virtual reality job interview tool (VR-JIT)	A training tool, interactive partner, progress monitoring system	VR-JIT plus services as usual. Participants practiced job interviews with a virtual interviewer named “Molly Porter”	10 h of VR-JIT over 2-month period, mean of 15.6 (SD = 2.9) interviews	10	MIRS, self-report scales for anxiety and self-efficacy	MIRS: The virtual reality intervention group showed a statistically significant increase in “Interviewee Performance” compared to control group	MIRS: improvement - Interviewee Performance domain	High acceptability
Horace H.S. Ip et al. (2018) (26)	Immersive Virtual Reality (projection-based half-CAVE system)	Assistant tool to provide simulated social scenarios	A session with three-stage structure: Briefing, VR-enabled Training, Debriefing	14 weeks (2 sessions per week)	4.7	PEP-3, ABAS-II, Faces Test & Eyes Test	PEP-3 Affective Expressions: The training group improved from 18.9 to 20.2 (p = 0.037). PEP-3 Social Reciprocity: The training group score increased from 20.2 to 21.8 (p < 0.001).	Improvement in social interaction and adaptation	High acceptability
Lingling Deng et al. (2022) (28)	Wearable sensors (Apple Watch, Arduino), SMRS app	Assistant Supplement, Progress Monitoring System, Adaptive Learning Tool	Session in the classrooms with a teacher and participant with an Apple Watch and GSR sensor. AI recommended physical or environmental adjustments	3 sessions for 30 minutes each (No-SMRS, SMRS session 1 and 2)	1	C-TRF, SUS, Heart rate, GSR, hand movements, and environmental data.	C-TRF: improvements in the ASD group pre- and post-intervention phase across all behavioral parameters (p < 0.001).	C-TRF: improvement in attention and stress	High acceptability
Voss, C. et al. (2019) (30)	Wearable Augmented Reality (Google Glass) paired with a smartphone application	Assistant Supplement, Progress Monitoring System, Adaptive Learning Tool	“Superpower Glass” intervention at home plus standard ABA therapy. Three activity modes: child elicits an emotion, caregiver acts out an emotion and unstructured social interaction	24 sessions (4 times per week for 6 weeks). The actual mean usage was 12.12 sessions	4	Primary: VABS-II, SRS-II, NEPSY-II, and EGG. Secondary: CBCL and VABS-II	Improvement in intervention group on the VABS-II socialization subdomain with a mean score increase of 4.58 points versus 1.05 points respectively (p = 0.005). Positive mean treatment	Results for the SRS-II, NEPSY-II, and EGG not significant	Eight withdrawals
Harini Atturu et al. (2024) (31)	The CognitiveBotics platform	Assistant Supplement, Progress Monitoring System, Adaptive Learning Tool	The four primary learning modalities: Audiovisual Stimulation, Chatbot, AI Interactive Games, Parent Training Videos	One session for 20 minutes, 3 sessions/day for 365 days	365	CARS, VSMS, DST, REEL	The AI-driven adaptive learning platform generated improvements from baseline to the 12-month mark, showing a highly significant increase in mean CARS scores (p < 0.001).	CARS: improvements in social, developement and expressive language age. Improvements in social and developement quotient	High acceptability
Brian Scassellati et al. (2018) (35)	Socially assistive humanoid robot	Assistant Supplement, Behavioral Monitoring System, Adaptive Learning Tool	A home-based program with a three-way interaction between the child, a caregiver, and the robot. Games helping social and emotional understanding, perspective-taking	23.25 sessions in 30 days	12.7	Validated joint attention assessment of Bean and Eigsti	The mean score increased from 15.67 in the baseline test to 20.89 after the intervention.	Improvement in joint attention skills in the absence of the robot. Increase in child’s social skills and increased eye contact	High acceptability
Eva Yin-han Chung (2019) (37)	Humanoid NAO robot	Assistant Supplement, Behavioral Monitoring System, Adaptive Learning Tool	The standardized activity protocol with 5 categories: Greetings, Interactive Games, Singing and Dancing, Storytelling, Round up	30 minutes session once a week for 12 weeks	6	SRS	Median scores the pre- (Pre) and post-intervention (End): Eye contact frequency: from 17.5 (Pre) to 41.5 (End). Verbal initiation frequency: from 3.0 (Pre) to 10.5 (End). Results showed significant differences between point 1 (Pre) to point 3 (End) for all variables.	ADOS: Improvements in communication and reciprocal social interaction	High acceptability
Eva Yin-han Chung et al. (2025) (38)	Humanoid NAO robot	Assistant Supplement, Behavioral Monitoring System, Adaptive Learning Tool	The standardized activity protocol with 5 categories: Greetings, Interactive Games, Singing and Dancing, Storytelling, Round up	35 minutes session once a week for 12 weeks	7	ADOS-2, SRS	ADOS-2: difference between groups (p < 0.001), very large effect size η2 = 0.612. SRS: difference between groups (p = 0.006), large effect size (η2 = 0.166)	ADOS: Improvements in communication and reciprocal social interaction. SRS: The robotic intervention group outperformed the control group	N/A

ABAS-II, Adaptive Behavior Assessment System, Second Edition; ADOS, Autism Diagnostic Observation Schedule, Second Edition; AI, Artificial Intelligence; CBCL, Child Behavior Checklist; C-TRF, Caregiver-Teacher Report Form; DST, Developmental Screening Test; EGG, Emotion Guessing Game; GSR, Galvanic Skin Response; MIRS, Mock Interview Rating Scale; NEPSY-II, Developmental Neuropsychological Assessment, Second Edition; PEP-3, Psychoeducational Profile, Third Edition; REEL, Receptive Expressive Emergent Language Test; SG, Study Group; SMRS, Sensory Management Recommendation System; SRS, Social Responsiveness Scale; SRS-T, Parent-Perceived Social Responsiveness; SUS, System Usability Scale; VABS-II, Vineland Adaptive Behavior Scales; VR, Virtual Reality; VSMS, Vineland Social Maturity Scale.

**Table 4c:** Emotion domain of reviewed studies.

Study	Type of technology	Role of AI	Intervention - SG	Duration of intervention	Total duration of intervention (hours) per participant	Assessments	Main results	Main outcomes	Acceptability of used system
Horace H.S et al. (2018) (26)	Immersive Virtual Reality (projection-based half-CAVE system)	Assistant tool to provide simulated social scenarios	Each session followed a three-stage structure: Briefing, VR-enabled Training, Debriefing	14 weeks (2 sessions per week)	approximately 5	PEP-3, ABAS-II, Faces Test & Eyes Test	PEP-3 Affective Expressions: The training group improved from 18.9 to 20.2 (p = 0.037). Social Reciprocity: The training group score increased from 20.2 to 21.8 (p < 0.001).	Improvements in emotional expression and regulation	High acceptibility
Voss C. et al. (2019) (30)	Wearable Augmented Reality (Google Glass) with a mobile app	Assistant Supplement, Behavioral Monitoring System, Adaptive Learning Tool	“Superpower Glass” intervention at home plus standard ABA therapy. Three activity modes: child elicits an emotion, caregiver acts out an emotion and unstructured social interaction	24 sessions (4 times per week for 6 weeks). The actual mean usage was 12.12 sessions	4	Primary: VABS-II Socialization, SRS-II, NEPSY-II, and EGG. Secondary: CBCL and VABS-II	The intervention group showed an improvement on the VABS-II socialization subdomain with a mean score increase of 4.58 points versus 1.05 points respectively (p = 0.005).	No improvements in emotion recognition	Eight withdrawals
Piana S. et al. (2022) (32)	Kinect v2, EyesWeb XMI Platform	Assistant Supplement, Behavioral Monitoring System, Adaptive Learning Tool	Training program utilizing the “Guess the Emotion”. Game with two specific tasks: Recognition Task and Expression Task	Approximately 4 to 6 weeks (2-3 sessions a week)	3.3	Leiter International Performance Scale, Wechsler Intelligence Scale for Children, TEC	TEC: pre-to-post intervention improvement within the experimental group (p = 0.036, partial η² = 0.402). Mean TEC clinical scores improved from 6.50 to 7.40.	Improvements in emotion recognition	High acceptibility
Fan M. et al. (2025) (33)	Web-based interactive system.	Assistant Supplement, Behavioral Monitoring System, Adaptive Learning Tool	Context-based narrative animations with five levels of interactive games: matching, mimicking, and spontaneously producing facial expressions	3 to 7 sessions (one session 30 minutes)	range 1,5-3,5	Assesment of facial expression recognition and expression, a five-point scale assessment sheet for recording emotional performance	An increase in mean facial expression recognition scores using static cards from 2.19 to 3.07 (p<0.01) and in star acquisition rates for facial expression production from 0.15 to 0.60 (p<0.001).	Improvements in recognizing facial expressions	High acceptibility
Guobin Wan et al. (2022) (34)	A camera-equipped computer with the proprietary FECTS system	Assistant Supplement, Behavioral Monitoring System, Adaptive Learning Tool	The training tasks as mini games. System included: Baseline Test, Emotion Matching, Emotion Inference, Emotion Imitation	4 days (one session pre day)	approximately 1	System data, interaction sheets manually recorded by an observer	The system produced pre-to-post clinical improvement in emotion identification and landmark accuracy scores among children in the experimental group.	Improvements in emotional expression and recognition	High acceptibility
Flavia Marino et al. (2020) (36)	Humanoid social robot - NAO	Intervention mediator and “co-therapist”	Robot-Assisted Cognitive Behavioural Therapy (RA-CBT). The robot assisted the human clinician in delivering the protocol	10 weeks	15	TEC, ELT	TEC: Robot group showed a significant 59% improvement in total scores (p = 0.001). ELT: Robot group total scores increased by 48% (p = 0.001).	Improvements in emotional expression and regulation	High acceptibility

ABA, Applied Behavioral Analysis; ABAS-II, Adaptive Behavior Assessment System, Second Edition; AI, Artificial Intelligence; CBCL, Child Behavior Checklist; EGG, Emotion Guessing Game; ELT, Emotion Lexicon Test; FECTS, A Facial Emotion Cognition and Training System; NEPSY-II, Developmental Neuropsychological Assessment, Second Edition; PEP-3, Psychoeducational Profile, Third Edition; SG, Study Group; SRS-II, Social Responsiveness Scale; TEC, Test of Emotion Comprehension; VABS-II, Vineland Adaptive Behavior Scales; VR, Virtual Reality.

### 3.1 Social domains

Advanced technologies, including virtual reality for job interview simulations, are used to train social domains in people with ASD and immersive half-CAVE projection systems, which are safe places to practice real-life social settings (25, 26). These interventions use Google Glass, Apple Watches, and sensors to track emotions as they happen. This technology provides real-time data and sensory advice to help a person manage their emotional responses (28, 30, 31). These AI technologies can be grouped into two main types: CAI-style platforms and social humanoid robots (robots that look like people). A common example of this is the NAO robot (27, 31, 35, 38, 39)—become active partners of participants, instructors and mediators that will aid in training the participants’ social skill. In the context of social functioning, AI application aims to support the development of interpersonal and communication skills, including eyes contact, emotional recognition, joint attention, and reciprocal interaction. AI-driven systems are capable of continuously monitoring user behavior and adapting the difficulty, pace, and type of social stimuli in real time. Such adaptive and personalized environments may reduce anxiety associated with unpredictable social situations and facilitate repeated practice of social behaviors in controlled setting. Conducting behavioral analysis, these systems enable automated assessments of progress and adjustment of difficulty and task level based on the users (25, 31, 38, 39).

Studies reviewed showed very wide diversity with respect to time and intensity. The shortest protocols comprised three 30 min sessions only (31), while shorter-term models adopt highly condensed activities; 23.25 sessions in 30 days are reached (31). Therapeutic activities ranged in intensity and usually spanned between 6 to 14 weeks. The average total training time in shorter cycles was about 10h (25) or 12 sessions in practice on average (30). In longer, systemically applied interventions, one session per week (30–35 minutes) (37–39), two meetings per week (26) and the longest model, based on a 12-month progress monitoring framework, with checkpoints at halfway point and the culmination of the project (31).

Difficulty making eye contact and reading gaze is a fact of life in individuals with ASD (26, 30). Findings show that humanoid robots, as is the case for NAO, drastically increase the amount of eye contact (37) so that joint attention, which is an important precursor to higher-order social interaction skills (35), can be encouraged. Robotic and AI-based interventions implemented at the home (e.g., CognitiveBotics) enhance visual attention and the sharing of attention by adults substantially and sustain their effectiveness outside the clinical setting (31, 35). In these home-based scenarios, AI algorithms act as a continuous, objective monitoring tool that filters out environmental noise, ensuring that the tracking of social responsiveness remains accurate in non-controlled, everyday environments. Family describes a sudden surge of eye contact in everyday life after robotic intervention (35).

Several trials using standardized instruments such as ADOS have shown statistically significant advancements in communication and reciprocal social interaction subscales (37–39). Mean CARS scores decreased as well (31), and there was an increase in SRS scores, which are measures of social responsiveness (37, 39). There was an increase in social and developmental age, and the highest trend was in expressive language. Both social and developmental quotients also rose, and joint attention skills increased in adults to adults in a robotic environment (35).

However, with these positive results there are some subgroups that were not significantly improved. Results of NEPSY-II and Emotion Guessing Game (EGG) tests did not reach the threshold of statistical significance compared to control groups (30). Likewise, a few studies reported no difference between restricted and repetitive behaviors (37, 39) and in one instance, there was no difference in the SRS scores of the robot-assisted group versus the human-instructed group (38).

### 3.2 Cognitive domains

In the cognitive intervention domain, reviewed studies applied AI-driven technologies such as Immersive Reality System, Wearable Sensors, Large Language Models (LLMs) such as ChatGPT, and Computer-Assisted Intervention (CAI). Research within the cognitive domain revealed significant methodological heterogeneity, which makes the comparison of the results more difficult. This diversity was visible in the selection of technologies, and the evaluation of specific cognitive functions. Despite the diverse measured parameters, a statistically significant improvement in the intervened cognitive functions was observed across all reviewed cases.

The role of AI in the discussed research protocols focused on assistant functions and adaptive progress monitoring. A notable implementation is the Sensory Management Recommendation System (SMRS) mobile application developed by Deng et al. (28). This system facilitates the synthesis of individual sensory profiles with real-time environmental data acquisition via integrated sensors. Central to the SMRS architecture is a Fuzzy Logic-based decision-making module, which quantifies risk levels to deliver precise, automated sensory management strategies specifically important for children with ASD. Significant differences were reported regarding the duration of the intervention, which ranged from 1 to 365 hours; however, two studies showed convergence within the 6–12 hour range. Regardless of the session duration, the results indicate high efficacy in improving the applied metrics, while maintaining a high level of technology acceptance and participant engagement.

### 3.3 Emotion domains

Types of technology supported by Artificial Intelligence, which aim to improve emotion recognition and expression in autistic individuals, vary from immersive virtual reality systems, camera-equipped computers to humanoid social robots. Research into AI-driven autism interventions demonstrates a shared objective of improving emotion recognition and expression through diverse technologies. Horace H.S. and Voss C (26, 30). established immersive VR and AR tools like “Superpower Glass” as adaptive assistants to reinforce facial engagement. In contrast, Marino et al. and Guobin Wan et al. focused on structured instruction using humanoid robots and the FECTS system to teach emotion labelling and context associations (34, 36). Finally, Piana et al. and Fan et al. utilized interactive gaming to train affective expression (32, 33). Despite these different delivery methods, all studies leveraged AI to automate behavioral inference and provide real-time feedback, transforming these emotion-focused exercises into responsive, adaptive training.

The dosage and duration of the interventions exhibit significant variability, ranging from intensive four-day protocols to longitudinal 14-week programs. Moreover, interventions involving immersive virtual reality or humanoid robotics applied a higher total hour-per-participant ratio compared to game-based training. Marino et al. reported the highest cumulative exposure, totaling 15 hours of robot-assisted cognitive behavioral therapy over 10 weeks (34). Conversely, Wan et al. utilized the most condensed timeframe, employing a four-day consecutive protocol of gamified emotional intelligence modules.

The assessment methodologies differ from standardized instruments such as the Test of Emotion Comprehension (TEC) and the Psychoeducational Profile, Third Edition (PEP-3), alongside specialized metrics like the Emotion Guessing Game (EEG). These tools were utilized to evaluate emotional expression, regulation, and social reciprocity. Research into these AI-driven interventions reveals mixed but promising results across various assessment domains. Gamified platforms showed the most consistent success, such as significant improvement in facial expression recognition and participant-reported comfort (32, 33, 36). In contrast, wearable and immersive platforms displayed more specialized outcomes: while VR successfully enhanced emotional regulation in social simulations, the “Superpower Glass” AR system failed to outperform control groups in emotion recognition tasks. Similarly, Horace et al. found that while their intervention significantly improved affective expression and social reciprocity (as measured by the PEP-3), Eyes and Face Tests did not report statistically significant improvements (26).

Ultimately, while AI-integrated tools consistently boost social engagement and expression confidence, their effectiveness in improving specific recognition accuracy varies significantly depending on the delivery platform and the assessment of metrics applied in presented interventions.

## 4 Discussion

This systematic review demonstrates that the implementation of Artificial Intelligence (AI) in the therapeutic process for individuals on the autism spectrum (ASD) can significantly enhance the effectiveness of interventions (40). The analysis of the social, cognitive, and emotion domains reveals that Artificial Intelligence is no longer just a peripheral tool; it has become an adaptive moderator of the therapeutic process, providing support (40, 41). However, it is important to distinguish between AI itself and the broader technological platforms used in the analyzed studies. While several interventions employed VR, AR, robotics, or wearable devices as delivery systems, the AI component specifically referred to adaptive algorithms, machine learning-based behavioral analysis, predictive models, or decision-support systems capable of modifying therapeutic responses in real time. In many cases, the therapeutic benefit likely resulted from the interaction between the technological interface and the AI-driven personalization mechanisms rather than from the digital platform alone. The ability of AI to effectively adapt its technical capabilities to the learning profiles of individuals with ASD presents a novel pathway for personalized, scalable, low-anxiety interventions through the dynamic adjustment of task difficulty (42, 43). This process promoted higher participant engagement and led to measurable therapeutic advances (44). In addition, AI systems had an advanced ability to analyze multidimensional behavior — such as through eye-tracking — enabling the identification of subtle changes often imperceptible to standard human observation (45).

A core strength of AI-driven interventions is the ability to provide real-time adaptation. Unlike traditional manualized therapies, AI platforms (such as CAI and social robotics like NAO) can dynamically adjust the difficulty of social or cognitive tasks based on the user’s immediate performance (35). This creates an optimal and comfortable environment, preventing the frustration often associated with social learning in ASD. The results of these studies suggest that AI-enhanced VR, AR, and robotic systems may act as social buffers. The unpredictability of human social cues can be overwhelming for many individuals with ASD due to a fact that everyday social interactions involve the rapid and simultaneous decoding of different modes of communication such as microexpressions, body language, culture context and different vocal intonations. People with ASD often struggle with sensory processing and cognitive flexibility, which makes the continuous exposure to this complex and unpredictable stream of stimuli cause sensory and cognitive overload. Additionally, the difficulties with making accurate predictions about the behaviors of other people can provoke social anxiety. Luckily, AI technology provides a simplified and predictable interface, which significantly reduces cognitive load for the user. It eliminates the extraneous social cues and creates an environment with highly controlled and predictable interactions where the user has enough time and mental space to process the information. Significant progress on subscales such as the ADOS and SRS suggests that the skills developed in the “safe” digital environments—eye contact, joint attention (46, 47). This indicates that AI acts as a scaffolding mechanism rather than a replacement for human contact. The integration of wearable sensors (Apple Watch, Arduino) and Fuzzy Logic-based systems (e.g., SMRS) represents a shift toward data-driven therapy. By quantifying sensory risk levels and providing real-time cues, AI assists in emotional regulation before a behavioral meltdown occurs. This proactive approach is a significant advancement over reactive traditional methods (48).

At the same time, the growing integration of AI into ASD therapy raises important ethical and practical concerns (49, 50). Many AI-based systems rely on continuous behavioral monitoring, facial analysis, eye-tracking, or biometric data collection, creating potential risks related to privacy, data security, and informed consent, particularly in pediatric populations (50, 51). Another concern involves algorithmic bias, as AI systems trained predominantly on male or culturally homogeneous cohorts may fail to adequately recognize diverse ASD presentations, especially among females and underrepresented populations (52, 52). In addition, excessive reliance on automated systems could unintentionally reduce opportunities for natural human interaction if AI tools are treated as substitutes rather than supportive adjuncts to therapist-led care (49, 50). These issues highlight the need for transparent algorithm development, clinical oversight, and ethical guidelines governing the implementation of AI in autism interventions (50).

Despite these findings, the analyzed material is characterized by significant heterogeneity, which limits the extrapolation of conclusions to the general population. Discrepancies primarily concerned with methodological approaches, the types of devices utilized, and the measurement tools employed. A substantial confounding factor was the wide variance in intervention duration (ranging from 1 to 365 hours), although in most studies, the median exposure fluctuated between 4 and 12 hours. Methodological analysis revealed several deficits in the research structure of many papers. While AI tools (like apps and adaptive learning systems) show immediate promise, there is very little data on whether these skills generalize real-world environments over months or years. Oyeyemi Patricia Adako et al. emphasize that future research must move beyond “proof of concept” pilots to long-term impact studies to determine lasting effects when it comes to applying AI interventions in autism education (53). Moreover, a frequent shortcoming was the absence of control groups, which precludes the precise isolation of the AI-based intervention effect from other variables. However, only one control group consisted of typically developed children. Furthermore, the studies were characterized by relatively small sample sizes (often N < 20). These findings are consistent with previous reviews that also underscore significant methodological constraints inherent in the existing research (46, 54).

A pronounced gender imbalance was also noted; males dominated most cohorts, and in some instances, females were entirely excluded from the analysis. This marked gender disproportion constitutes a significant barrier to the interpretation of the data. The male dominance in AI research reflects a broader issue resulting from the lower diagnostic rates in women and their distinct symptomatic profiles as emphasized in various studies (55, 56). The lack of female representation prevents an assessment of whether AI algorithms are equally effective in recognizing subtle social deficits, which in women are often subject to “social camouflaging” (57).

Another key limitation is the lack of representation of the adult population (no study reported a mean age exceeding 18 years). However, the therapeutic needs of adults with ASD—often diagnosed only in adulthood—differ from those of children, encompassing domains such as vocational independence, navigating complex social structures, and household management. The study by Gotham et al. emphasizes the necessity of developing interventions specifically tailored to the challenges faced by autistic adults (58). This restricts the ability to draw conclusions regarding the effectiveness of AI-based interventions in this demographic. It is also suggested that cultural context has a significant impact on the acceptability and effectiveness of AI-based technologies (59). The strong geographical concentration of projects (primarily the USA and China) must be noted, which limits inferences regarding the effectiveness of AI in other cultural contexts.

There is an urgent need for randomized controlled trials (RCTs) that directly compare the effectiveness of conventional therapy with AI-assisted interventions. Future research should prioritize the use of AI to identify markers of camouflaging and to support therapy tailored to the distinct symptomatic phenotype observed in women. Another important research direction is the implementation of AI tools in the treatment of adults, particularly given the increasing number of late-age diagnoses. Further studies should also explore the use of AI to enhance the daily functioning of adults, including in professional environments, such as through AI-based applications that support task planning or facilitate communication in vocational settings. In summary, the application of AI in autism spectrum disorder (ASD) therapy demonstrates significant potential, especially in behavioral monitoring and the individualization of learning processes. The advancement of this technology may increase access to therapy and provide support not only for therapists but also for teachers and parents in both home and educational contexts.

### 4.1 Ethical concerns

With the implementation of presented AI-based interventions into healthcare, concerns of data privacy and understanding must be addressed. For instance, treatments often include therapy transcripts, video recordings, and other clinical history that must be monitored carefully and protected. These systems need to robustly follow the legal framework of the Health Insurance Portability and Accountability Act (HIPAA) to keep them from being accessed by outside parties and to ensure that caregivers and participants understand how their information is being accessed and used (60).

### 4.2 Practical recommendations

In clinical practice AI and robotics may be presented as initial “ice breakers” or interventional tools to establish foundational behavioral skills including eye contact. After this, a gradual transition to human-led sessions may promote a wider social integration. With heterogeneous data in mind, the results suggest that shorter, higher intensity protocols (for example, 10–12 total hours) may be as effective as longer, lower frequency strategies. As a result, it may be more effective to optimize intervention intensity based on individual patient tolerance rather than to simply extend the intervention duration. Furthermore, wearable technology applied for measuring exposure to sensory load may provide useful, AI-enabled information. The integration of wearable applications may also help therapists in managing overstimulation, by anticipating and responding to it.

In order to navigate the methodological heterogeneity presented in this review, future research could seek a direction toward a more standardized assessment model. By the combination of tests—e.g., the ADOS-2 with the AI-generated metrics—the feasibility and reliability of future meta-analyses may increase. Designing tools with built-in transitional modules may help shift the user’s interaction from a digital or robotic interface to a human peer.

For home-based interventions the use of accessible AI platforms (e.g., CognitiveBotics) might be a feasible option in terms of continuity of care. The utilization of such platforms may potentially help support the sustainability and consistency of these longer-term, 12-month monitoring approaches (31).

## 5 Conclusion

This systematic review indicates that Artificial Intelligence offers an opportunity as an adaptive real-time scaffolding tool in ASD interventions across social, cognitive and emotional domains. By providing predictable interactions and adjusting task difficulty, AI-driven platforms help to reduce cognitive load and enhance therapeutic engagement. Nevertheless, the existing research has several limitations, including diverse methodology, number of participants and the lack of studies involving both women and adults with ASD. In order to bring AI into the practice of clinical use, more research is needed that would involve large randomized controlled trials (RCTs) while resolving challenges of ethical nature.

## Data Availability

The original contributions presented in the study are included in the article/supplementary material, further inquiries can be directed to the corresponding author/s.
